# OAS3 Deubiquitination Due to E3 Ligase TRIM21 Downregulation Promotes Epithelial Cell Apoptosis and Drives Sepsis-induced Acute Lung Injury

**DOI:** 10.7150/ijbs.96089

**Published:** 2024-10-14

**Authors:** Zhenfeng Chen, Bingqi Lin, Xiaodan Yao, Yihang Fang, Jinlian Liu, Ke Song, Lina Tuolihong, Zirui Zuo, Qi He, Xiaoxia Huang, Zhuanhua Liu, Qiaobing Huang, Qiulin Xu, Zhifeng Liu, Xiaohua Guo

**Affiliations:** 1 Guangdong Provincial Key Laboratory of Cardiac Function and Microcirculation, National Experimental Education Demonstration Center for Basic Medical Sciences, Department of Pathophysiology, School of Basic Medical Sciences, Southern Medical University, Guangzhou, 510515, China.; 2 Department of Medical Critical Care Medicine, General Hospital of Southern Theatre Command of PLA, Guangdong Branch Center, National Clinical Research Center for Geriatric Diseases (Chinese PLA General Hospital), Guangzhou, 510515, China.; 3 Department of Intensive Care Unit, Guangdong Provincial People's Hospital, Guangdong Academy of Medical Science, Southern Medical University, Guangzhou, 510515, China.

**Keywords:** OAS3, Ubiquitination, TRIM21, Apoptosis, Sepsis, Acute lung injury

## Abstract

Patients with sepsis-induced acute lung injury (SALI) show a high mortality rate, and there is no effective treatment in the clinic for SALI but only symptomatic treatment as an option. Therefore, searching for effective targets is critical for the management of SALI. Ubiquitination is an essential post-translational protein modification involved in most pathophysiological processes. However, the relationship between ubiquitination and SALI remains largely unclear. In this study, we examined the ubiquitination modification changes in SALI, identified oligoadenylate synthetase 3 (OAS3) as a key candidate accounting for SALI from integrative multi-omics analysis and confirmed its role in promoting SALI and cell apoptosis in an animal model of cecal ligation and puncture-treated mice and a cellular model of LPS-treated MLE12 cells. Mechanistically, downregulation of E3 ligase TRIM21 mediates the reduction of OAS3 K48-linked polyubiquitination at the K1079 site in lung epithelial cells of a septic model, which leads to the increase of OAS3 protein level in a proteasomal-dependent manner. The upregulated OAS3 promotes epithelial cell apoptosis through its downstream effector molecule, RNase L. In summary, these findings unveil a previously unappreciated role of OAS3 ubiquitination in SALI and offer a promising perspective for further understanding the development of sepsis and potential therapeutic target for the treatment of SALI.

## Introduction

Sepsis, a disease characterized by organ dysfunction resulting from the dysregulation of host inflammatory responses to systemic infection, is a worldwide healthcare concern^1^,^2^. The lung is the most susceptible target organ among the many organs involved in sepsis^3^. Patients with sepsis are very prone to acute lung injury (ALI), and the incidence and mortality of patients with sepsis-induced acute lung injury (SALI) are as high as 40.2% and 30%-85%, respectively, which is an important subject concerned by intensive care medicine and needs to be solved urgently^4^. However, there is no effective treatment in the clinic for SALI but only symptomatic treatment. Consequently, identifying effective targets and therapeutic strategies is crucial for the management of SALI.

Ubiquitination is a common and important post-translational modification (PTM) that exists widely in eukaryotic cells, requiring the sequential activity of activating (E1), conjugating (E2) and ligating (E3) enzymes^5^,^6^. Ubiquitination serves a complicated regulatory function in sepsis, involving in NF-κB activation and the pathobiology of ARDS induced by septic shock^7^. It is also reported that the E3 ubiquitin ligase CHIP helps protect against sepsis-induced myocardial dysfunction by facilitating the ubiquitination and degradation of karyopherin-α2^8^. However, there are few studies systematically investigating the role of ubiquitination modification in sepsis through ubiquitination omics. The effect of protein ubiquitination on SALI remains to be thoroughly investigated.

OAS3 (2'-5'-oligoadenylate synthetase 3) is an enzyme activated by double-stranded RNA (dsRNA) and is essential in the innate immune response against viral infections. Upon binding dsRNA, OAS3 synthesizes 2'-5'-linked oligoadenylate (2-5A) and triggers the activation of RNase L (a pseudokinase-endoribonuclease) which exerts antiviral activity by cleaving single-stranded RNA (ssRNA) from virus reproduction as well as host cell such as mRNA to suppress viral and cellular protein synthesis^9^. Besides its antiviral functions, OAS3/RNase L has also been reported to participate in apoptosis^10^-^12^. However, it remains unclear whether OAS3/RNase L mediates apoptosis of lung epithelial cells during septic progression.

TRIM21, a member of the Tripartite motif-containing (TRIM) family, is characterized as an E3 ubiquitin ligase that catalyzes the ubiquitination of various substrates and targets them for degradation^13^-^15^, leading to numerous diseases such as inflammatory bowel disease, autoimmune diseases and cancer^14^,^16^,^17^. It is not clear whether OAS3 acts as a substrate for TRIM21. In addition, it is only reported that TRIM21 alleviates LPS-mediated inflammatory responses in lung endothelial cells within a murine model of ALI^18^, while its role in lung epithelial cells remains to be explored.

In this study, we examined the ubiquitination modification changes in SALI and identified OAS3 as a key candidate accounting for SALI from integrative multi-omics analysis. Further investigation proved that downregulation of E3 ligase TRIM21 mediates the reduction of OAS3 K48-linked polyubiquitination at the K1079 site in lung epithelial cells of a septic model, which leads to an increase in OAS3 protein level in a proteasomal-dependent manner. The upregulated OAS3 promotes epithelial cell apoptosis through its downstream effector molecule, RNase L. These findings offer a novel perspective for further understanding the progression of sepsis and identifying potential therapeutic targets for its treatment.

## Materials and Methods

### Reagents

Detailed information on key materials used in this study is presented in Table S1, including antibodies, recombinant DNA and chemicals.

### Sepsis model

Cecal ligation and puncture (CLP)-induced septic mouse model, a gold standard mouse model for pre-clinical sepsis research^19^, was employed and established as described before^20^. C57BL/6 mice aged 6-8 weeks were obtained from the Laboratory Animal Center of Southern Medical University. All animal experiments were performed under protocols approved by the Animal Care and Use Committee of Southern Medical University. Survival study with mice were monitored for 5 days after CLP operation. Mice that survived for more than 5 days were sacrificed by cervical dislocation on the fifth day after operation.

Lipopolysaccharide (LPS)-induced mouse lung epithelial (MLE12) cells were applied as the septic lung epithelial cell model. MLE12 cells were cultured in DMEM/F12 medium (Gibco) supplemented with 10% FBS (ExCell) and maintained in a 37℃ incubator containing 5% CO_2_. Although the septic cell model is still controversial, stimulation of cells with LPS is currently the most common method to simulate the cell circumstance of sepsis^21^.

### Multi-omics analysis

The multi-omics (transcriptome, proteome and ubiquitinome) analysis of lung tissue from Sham or CLP mice was conducted to identify changes in gene expression, protein abundance and ubiquitin modification associated with sepsis-induced acute lung injury. The several assays, including RNA extraction and analysis of transcriptome, protein preparation and analysis of proteome and ubiquitinome, and LC-MS analysis of IP samples, were performed with the technical assistance from Novogene Bio Co. Ltd. (Beijing, China) and PTM Bio Co. Ltd. (Hangzhou, China). The sequencing data generated from this study were deposited into the Sequence Read Archive (SRA) with the accession number “PRJNA1066427”.

### Statistical analysis

Data were analyzed by GraphPad Prism 8.0 software (GraphPad Software, Inc.) and presented as *Mean* ± *SD*. The “*n*” reported in each experiment represents a biological replicate. The sample size for each study was determined in accordance with the literature documentation of similar well-characterized experiments. A Student's *t* test (two-sided) and one-way ANOVA with Turkey post-hoc test were used to determine the statistical difference between two groups and multiple groups respectively. Survival analysis was performed through Kaplan-Meier analysis followed by log-rank tests. *P* value less than 0.05 was considered statistically significant.

## Results

### A Multi-omics approach identifies OAS3 as a possible candidate accounting for sepsis-induced acute lung injury

**Figure 1:**
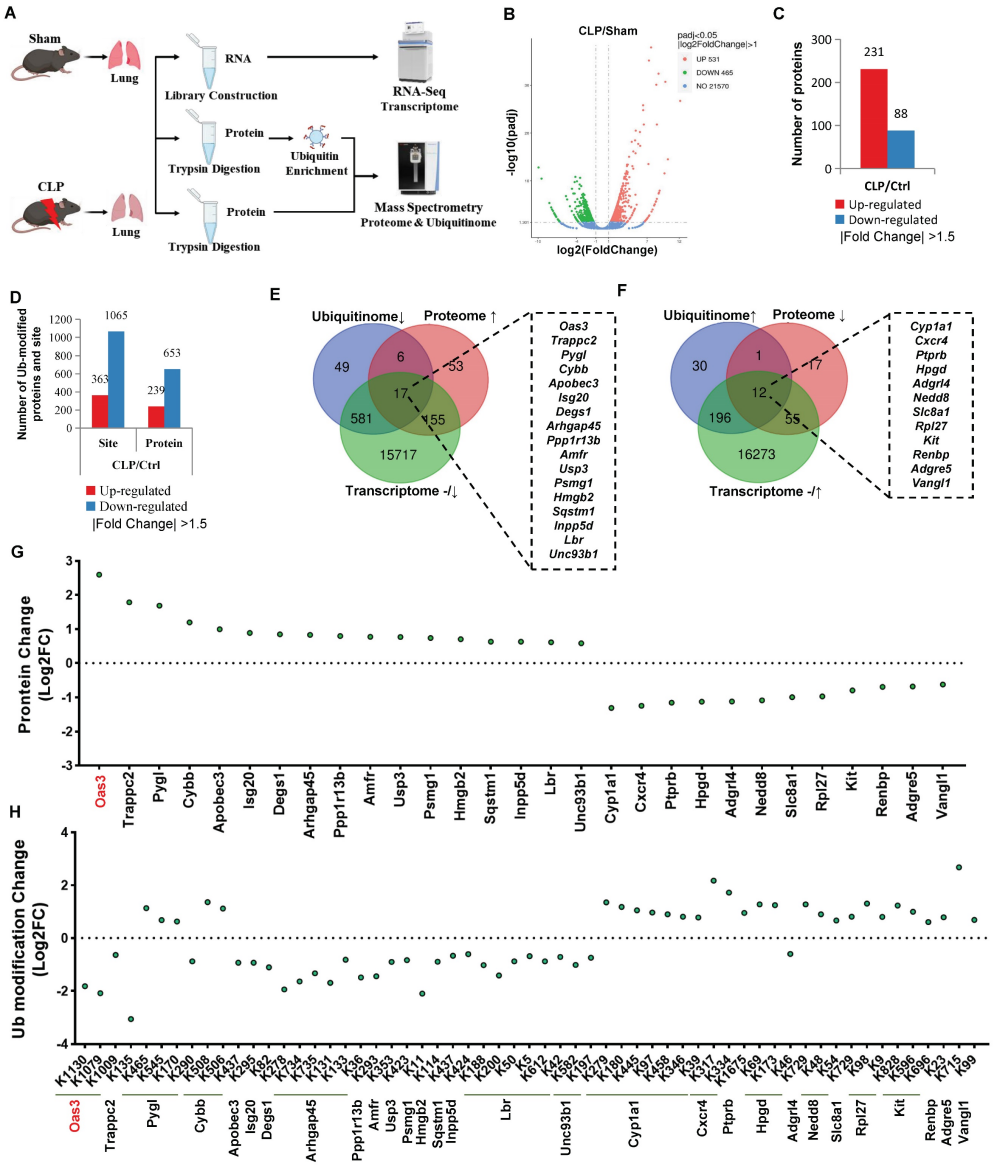
** A multi-omics approach identifies OAS3 as a candidate accounting for sepsis-induced lung injury. (A)** Schematic depiction of the workflow of multi-omics, including transcriptome, proteome and ubiquitinome.** (B)** Volcano plot of differentially expressed genes (DEGs) identified by transcriptome.** (C)** The number of differentially expressed proteins found in the proteome.** (D)** The number of differentially ubiquitin-modified proteins discovered by the ubiquitinome.** (E)** Venn diagram showing multi-omics screening results by Method 1.** (F)** Venn diagram showing multi-omics screening results by Method 2.** (G)** Analysis of the protein levels of the multi-omics intersection genes from the screening results.** (H)** Analysis of the ubiquitination modification level of the multi-omics intersection genes in the screening results.

To investigate the patterns of ubiquitination during the process of sepsis-induced acute lung injury (SALI), cecal ligation and puncture (CLP)-induced septic mouse model was established and then the lung tissue was harvested for global transcriptome, ubiquitinome and proteome analysis (Fig. 1A). After the genes (with a fold-change less than 2.0) and proteins and ubiquitination sites (with a fold-change less than 1.5) were filtered out, we observed that a total of 531 genes and 231 proteins were upregulated, while 465 genes and 88 proteins were downregulated (Fig. 1B-C, Table S2-3). Meanwhile, the ubiquitination of 363 lysine sites on 239 proteins was found to be increased and the ubiquitination of 1065 lysine sites on 653 proteins to be decreased (Fig. 1D, Table S4). Protein counts with differential ubiquitination significantly outnumbered the protein counts with differential expressions during CLP, suggesting that alterations in the post-translational modification (PTM) of the intracellular proteins are more adaptable and efficient than direct changes in the protein level in response to sepsis-induced lung injury. In addition, functional enrichment analysis of differentially ubiquitin-modified proteins also implied the key biological processes, cellular component, molecular function and KEGG pathway involved in this process (Fig. S1A-D).

Degradation-type ubiquitination modification is a main ubiquitination modification type participating in biological processes and we next employed two different screening methods of integrated multi-omics analysis to explore the potential candidates with degradation-type ubiquitination accounting for SALI. Method 1 refers to the intersection of decreased ubiquitination and increased protein level accompanied by an unaltered or decreased gene transcription level (Fig. S2A, C). On the contrary, Method 2 is defined as the intersection of increased ubiquitination, decreased protein level and an unaltered or increased gene transcription level (Fig. S2B, C). Respectively, 17 and 12 proteins conforming to the parameters of Methods 1 and 2 were found (Fig. 1E-F). Among them, OAS3 protein was altered to the greatest extent, with 3 ubiquitination sites, all of which changed in the direction of downregulation (Fig. 1G-H, Fig. S2D), suggesting that OAS3 is probably a key candidate protein accounting for SALI.

### The OAS3 K48-linked polyubiquitination decreases and the protein increases in mouse lung epithelial cells of sepsis model

**Figure 2:**
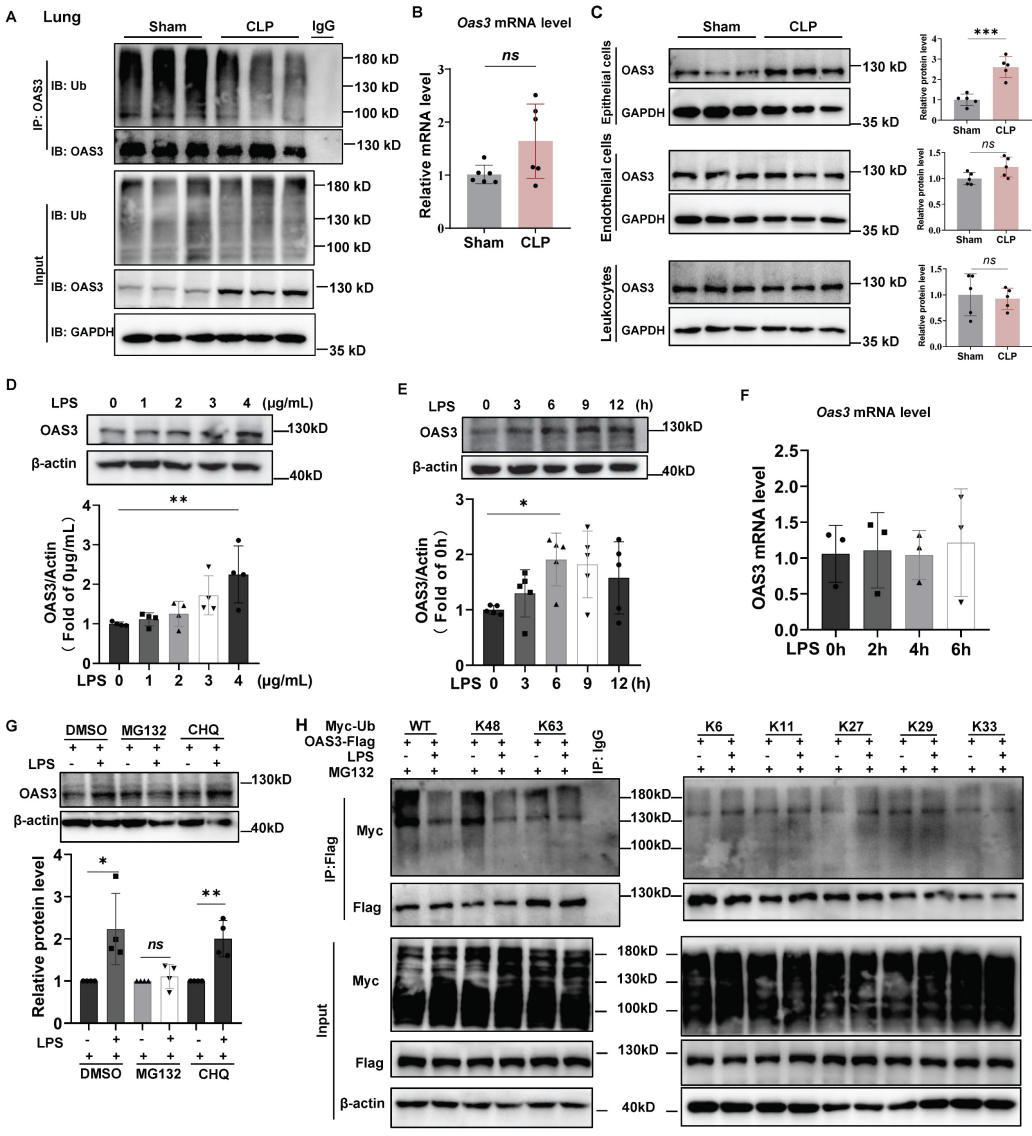
** The OAS3 K48-linked polyubiquitination decreases and the protein increases in mouse lung epithelial cells of sepsis model. (A)** Western blot analysis of OAS3 ubiquitination level and protein level in lung tissue of Sham and CLP mice (*n=*6).** (B)** qPCR analysis of *Oas3* mRNA level in lung tissue of Sham and CLP mice (*n=*6).** (C)** Western blot analysis of OAS3 protein level of epithelial cells, endothelial cells and leukocytes in the lung tissue of Sham and CLP mice (*n=*5).** (D)** Western blot analysis of OAS3 protein level in MLE12 cells treated with different concentrations of LPS for 6 h (*n=*4).** (E)** Western blot analysis of OAS3 protein level in MLE12 cells treated with LPS (4 μg/ml) for different times. (*n=*5).** (F)** qPCR analysis of *Oas3* mRNA level in MLE12 cells treated with LPS (4 μg/ml) for different times (*n=*3).** (G)** OAS3 protein level in MLE12 cells treated with LPS plus 10μM MG132 or 10μM chloroquine (CHQ) (*n=*4).** (H)** Ubiquitination of OAS3-Flag in MLE12 cells co-transfected with the indicated plasmids, followed by LPS plus MG132 treatment. Data are shown as *Mean* ± *SD*. *P<0.05, **P<0.01, ***P<0.001. *ns*: no significance.

Next, we verified the results of OAS3 change from multi-omics analysis. The ubiquitin-modified OAS3 level significantly decreased in the lung tissue of CLP group compared with that in Sham group (Fig. 2A). Meanwhile, the OAS3 protein level significantly increased, while its mRNA level had no significant change in the CLP group (Fig. 2A-B). These results were consistent with multi-omics results and indicated that the increase of OAS3 is regulated at the posttranscriptional level but not at the transcriptional level in the CLP model. We also found that the increase of OAS3 appeared in the epithelial cells of lung tissue but not in endothelial cells or leukocytes by flow sorting of these three cell types in the Sham and CLP groups (Fig. 2C, Fig. S3). These results were further confirmed in a cellular model of sepsis by treating mouse lung epithelial (MLE12) cells with LPS at the indicated concentration and time. The OAS3 protein level was elevated as LPS-treated concentration and time increased (Fig. 2D-E). However, the mRNA level of OAS3 was not increased correspondingly in LPS-treated MLE12 cells (Fig. 2F). This indicated that LPS also regulates the expression of OAS3 post-transcriptionally and that the increase in OAS3 protein level may be attributed to a decrease in its degradation. To test this hypothesis, we treated MLE12 cells with MG132 (a proteasome inhibitor) or CHQ (chloroquine, a lysosome inhibitor), respectively, and found that MG132, not CHQ, rescues the LPS-induced OAS3 upregulation (Fig. 2G). These results suggested that OAS3 is regulated in a degradative way, specifically in a proteasomal-dependent (not autophagy-dependent) manner under LPS condition.

Since different types of polyubiquitination linkages mediate distinct biological functions, we screened for potential lysine ubiquitination types of OAS3 by expressing flag-tagged OAS3 in MLE12 cells together with a series of ubiquitin mutants (K6, K11, K27, K29, K33, K48, and K63), all of which contained only one indicated lysine available for polyubiquitination linkage. Notably, LPS decreased K48-linked polyubiquitination of OAS3 but had no appreciable effect on the OAS3 ubiquitination of other linkage types (Fig. 2H), suggesting that LPS primarily inhibits the attachment of K48-linked polyubiquitination to OAS3.

### OAS3 accounts for sepsis-induced acute lung injury and lung epithelial cell apoptosis

**Figure 3:**
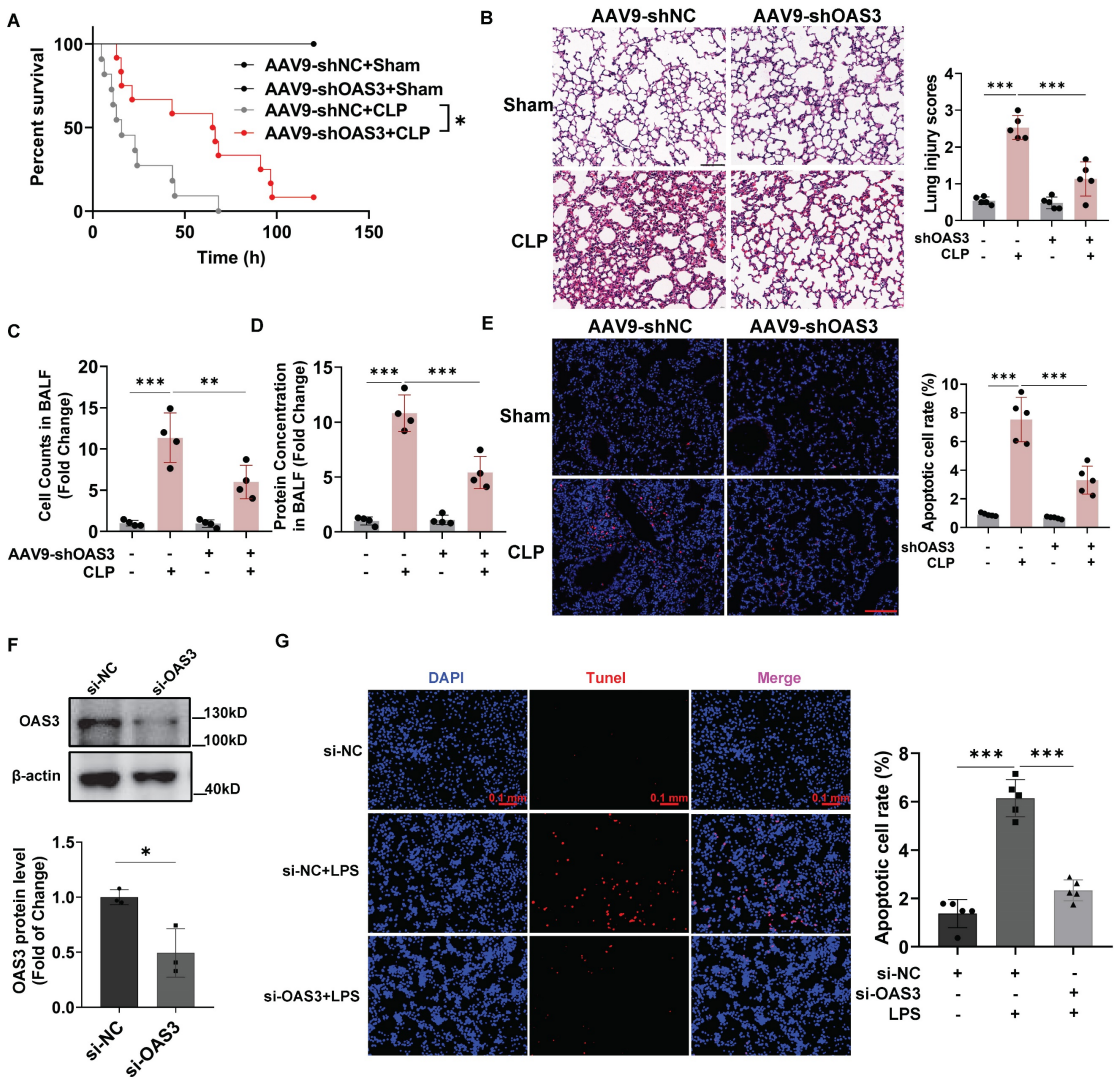
** OAS3 accounts for sepsis-induced acute lung injury and lung epithelial cell apoptosis. (A)** Kaplan-Meier survival analysis of Sham and CLP mice intratracheally injected with AAV9-shNC (negative control) or AAV9-shOAS3 (*n=*11).** (B)** Representative H&E staining and histological scores of lung sections in Sham and CLP mice intratracheally injected with NC-shRNA or OAS3-shRNA AAV9 (*n=*5, scale bar: 100 μm).** (C)** The cell number and** (D)** protein concentration in bronchoalveolar lavage fluid (BALF) of Sham and CLP mice intratracheally injected with NC-shRNA or OAS3-shRNA AAV9 (*n=*4).** (E)** TUNEL-positive cells (stained with Cy3, red) and nuclei (stained with DAPI, blue) in lung tissue of Sham and CLP mice intratracheally injected with NC-shRNA or OAS3-shRNA AAV9 (*n=*5, scale bar: 100 μm).** (F)** OAS3 protein level in MLE12 cells transfected with specific siRNA targeting OAS3 (*n=*3). **(G)** TUNEL-positive cells in MLE12 cells transfected with OAS3 siRNA followed by LPS treatment were observed and analyzed using a fluorescence microscope (Zeiss) (*n=*5, scale bar: 100 μm). Data are shown as *Mean* ± *SD*. *P<0.05, **P<0.01, ***P<0.001.

Next, we explored whether OAS3 upregulation is responsible for sepsis-induced acute lung injury (SALI) with lung epithelial cells-specifically expressed AAV9-shOAS3 (inserted with the SFTPC promoter element) intratracheally injected into the mouse lung. After validating the knock-down efficiency for OAS3 in lung epithelial cells (Fig. S4), we observed that AAV9-shOAS3 effectively prolonged the survival rate of mice with CLP operation compared with the AAV9-shNC treatment group (Fig. 3A). HE staining of lung tissue showed that AAV9-shOAS3 treatment attenuated the severe lung injury of septic mice characterized by alveolar septum thickening, neutrophil infiltration, hemorrhage, and edema (Fig. 3B). Besides, the AAV9-shOAS3-treated mouse showed a lower cell number and protein concentration in alveolar lavage fluid (BALF) than those in the AAV9-shNC treatment group after CLP procedure (Fig. 3C-D). These results demonstrated that OAS3 aggravates sepsis-induced acute lung injury.

Apoptosis is closely related to septic acute lung injury. In order to determine whether OAS3 could functionally influence sepsis-induced lung epithelial cell apoptosis, we analyzed the apoptosis levels *in vivo* and* in vitro* by TUNEL staining. We detected a marked decrease of apoptotic lung tissue cells that stained positive for TUNEL in the AAV9-shOAS3 group compared with the AAV9-shNC group in the setting of CLP model (Fig. 3E). Moreover, after verifying the interfere efficiency of OAS3 siRNA in MLE12 cells (Fig. 3F), we found that cells stained positive for TUNEL in the si-OAS3 group was also reduced compared with the si-NC group in the setting of LPS (Fig. 3G). These results suggested that OAS3 facilitates the apoptosis of the lung epithelial cells during sepsis.

### TRIM21 is identified as the E3 ligase for OAS3 K48-linked polyubiquitination

**Figure 4:**
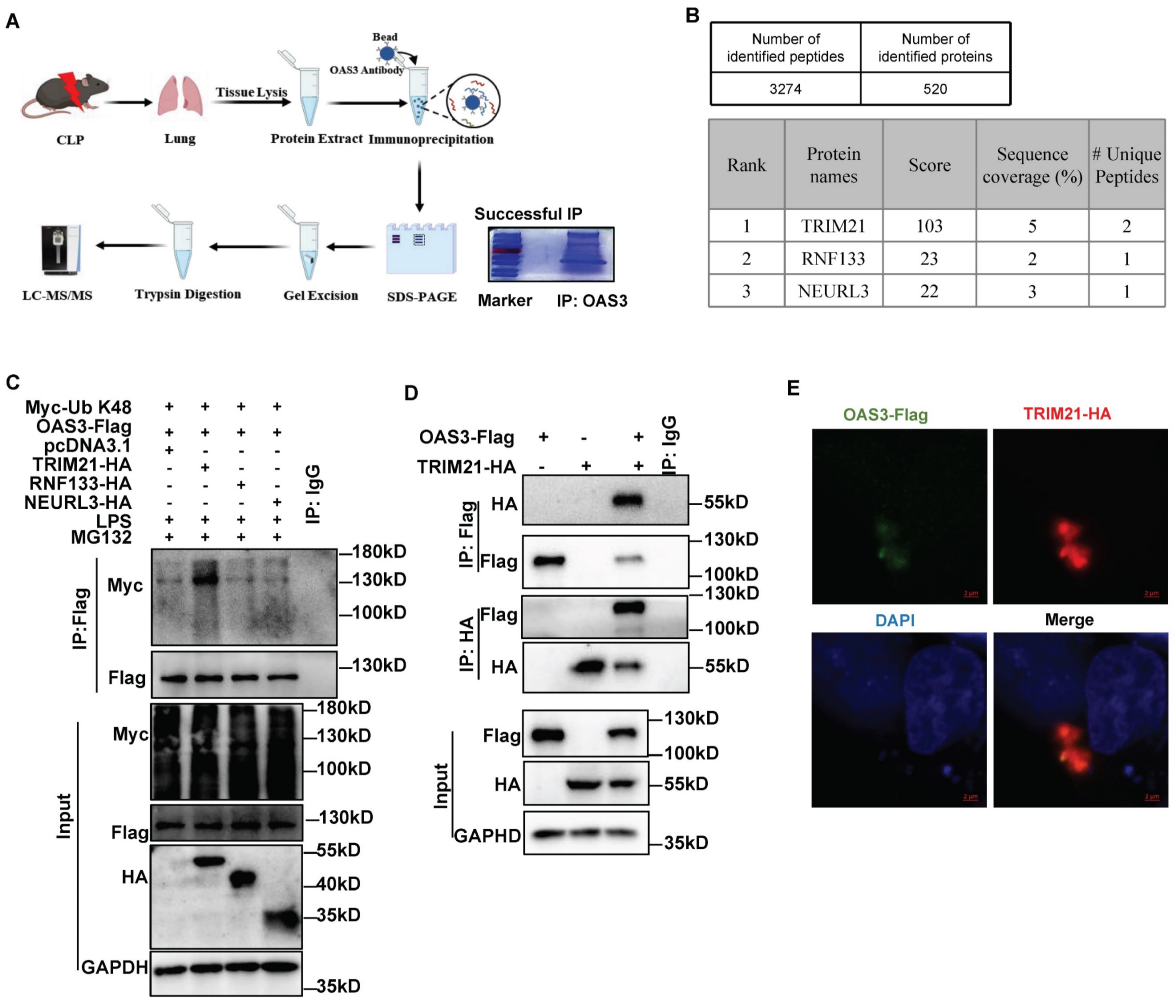
** TRIM21 is identified as the E3 ligase for OAS3 K48-linked polyubiquitination. (A)** Schematic diagram of the workflow of IP-mass spectrum (MS) to find E3 Ubiquitination or deubiquitinating enzyme interacted with OAS3.** (B)** The result of IP-MS. Proteins are ranked by score.** (C)** MLE12 cells were co-transfected with the indicated plasmids followed by LPS and MG132 treatment and Western blot analysis of the OAS3 ubiquitination level.** (D)** MLE12 cells were co-transfected with the OAS3-Flag and TRIM21-HA overexpression plasmids followed by a Co-IP assay to detect the interaction of the two proteins.** (E)** MLE12 cells were co-transfected with the OAS3-Flag and TRIM21-HA overexpression plasmids, and then the cells were stained with anti-Flag (green) and anti-HA (red) antibodies, as well as DAPI (blue) to detect the co-localization of OAS3 and TRIM21 by confocal microscopy (Zeiss) (scale bar: 2μm).

To investigate the E3 ubiquitin ligase or deubiquitinase (DUB) accounting for OAS3 ubiquitination, we screened the proteins that interact with OAS3 in the lung tissue of CLP mice through immunoprecipitation (IP)-MS (Fig. 4A). Finally, 520 proteins were identified, containing three E3 ubiquitin ligases and no DUBs (Fig. 4B, Table S5). MLE12 cells were overexpressed with these three E3 ubiquitin ligases individually, including TRIM21, RNF133 and NEURL3, along with the OAS3 and K48-Ub overexpression plasmids. It was shown that TRIM21 significantly increased K48-linked polyubiquitination of OAS3, while both RNF133 and NEURL3 had no such effect (Fig. 4C), indicating that TRIM21 is the potential E3 ligase for OAS3 K48-linked polyubiquitination. In addition, the interaction between OAS3 and TRIM21 was further confirmed by performing a Co-IP and immunofluorescence co-localization assay in MLE12 cells (Fig. 4D-E).

### Downregulation of TRIM21 increases OAS3 protein in sepsis

**Figure 5:**
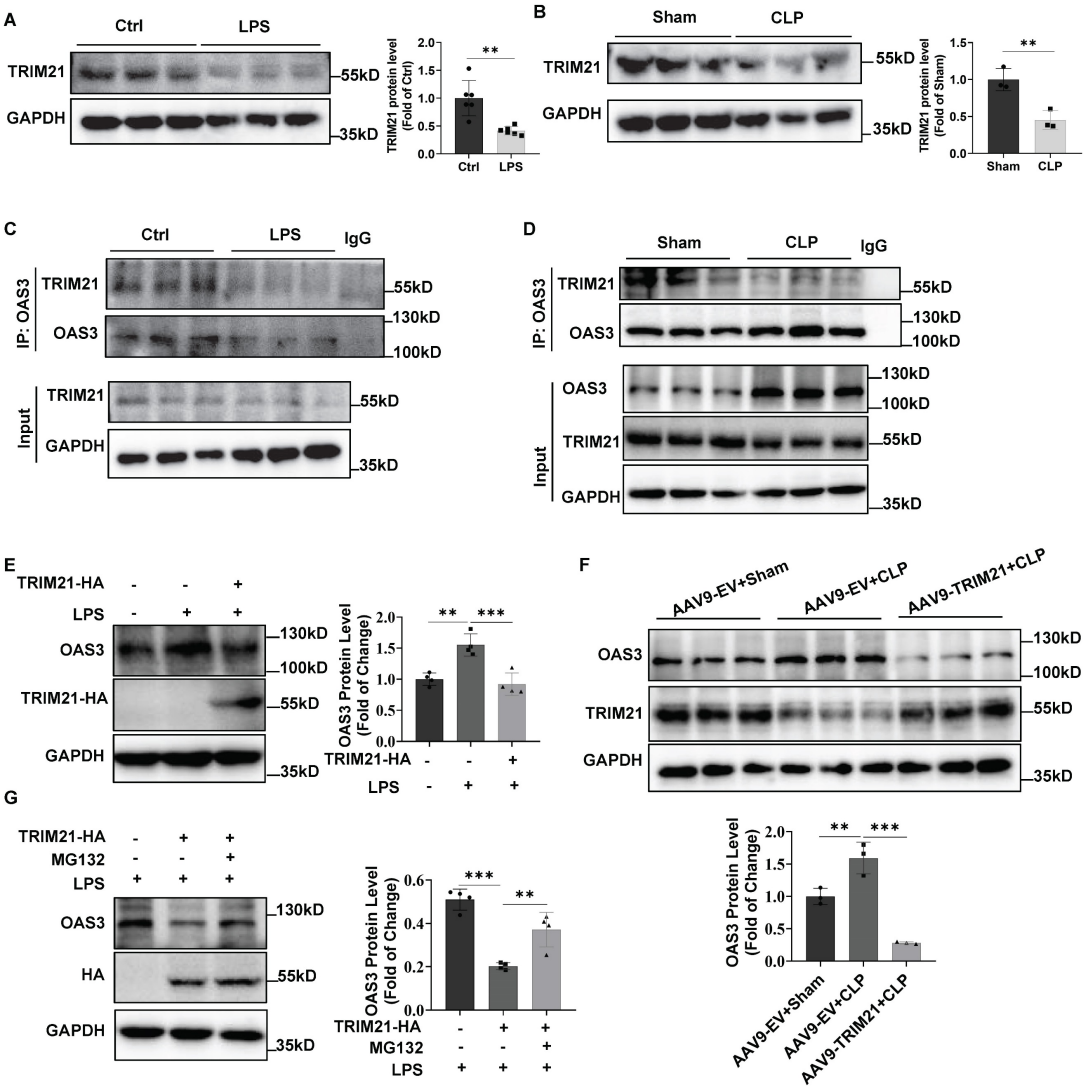
** Downregulation of TRIM21 increases OAS3 protein in sepsis. (A)** Western blot analysis of TRIM21 protein level in MLE12 cells treated with LPS. (*n=*6). **(B)** Western blot analysis of TRIM21 protein level in lung tissue of Sham and CLP mice. (*n=*3).** (C)** MLE12 cells were treated with or without LPS and then the interaction of OAS3 and TRIM21 was measured by Co-IP assay.** (D)** Co-IP assay analysis of the interaction between OAS3 and TRIM21 in lung tissue of Sham and CLP mice. (*n=*3).** (E)** Western blot analysis of OAS3 protein in MLE12 cells transfected with the TRIM21 overexpression plasmids followed by LPS treatment. (*n=*4).** (F)** Western blot analysis of OAS3 protein in lung tissue of mice intratracheally injected with the empty vector (EV) or TRIM21 overexpression AAV9 followed by CLP operation (*n=*3).** (G)** After being transfected with the TRIM21 overexpression plasmid, MLE12 cells were treated with or without MG132 plus LPS treatment, and then the OAS3 protein level was detected by Western blotting (*n=*4). Data are shown as *Mean* ± *SD*. **P<0.01, ***P<0.001.

Next, we detected TRIM21 expression in LPS-treated MLE12 cells and found that LPS significantly downregulated TRIM21 expression (Fig. 5A). *In vivo*, TRIM21 protein level also decreased in the lung tissue of CLP mice (Fig. 5B), which was consistent with the result of the cellular LPS model. Further, the Co-IP result showed that the interaction between OAS3 and TRIM21 was significantly reduced in MLE12 cells in response to LPS treatment (Fig. 5C) or in lung tissue of CLP mice (Fig. 5D), suggesting that the impaired interaction between OAS3 and TRIM21 may result from the downregulation of TRIM21 expression in response to sepsis. Notably, overexpression of TRIM21 in MLE12 cells or mice reversed the sepsis-induced increase in OAS3 protein level (Fig. 5E-F). Moreover, proteasome inhibitor MG132 attenuated TRIM21-mediated OAS3 reduction in the setting of LPS (Fig. 5G). These results indicated that sepsis increases OAS3 protein levels by downregulating TRIM21 expression in a proteasomal-dependent way.

### TRIM21 mediates OAS3 ubiquitination at K1079 under LPS treatment

**Figure 6:**
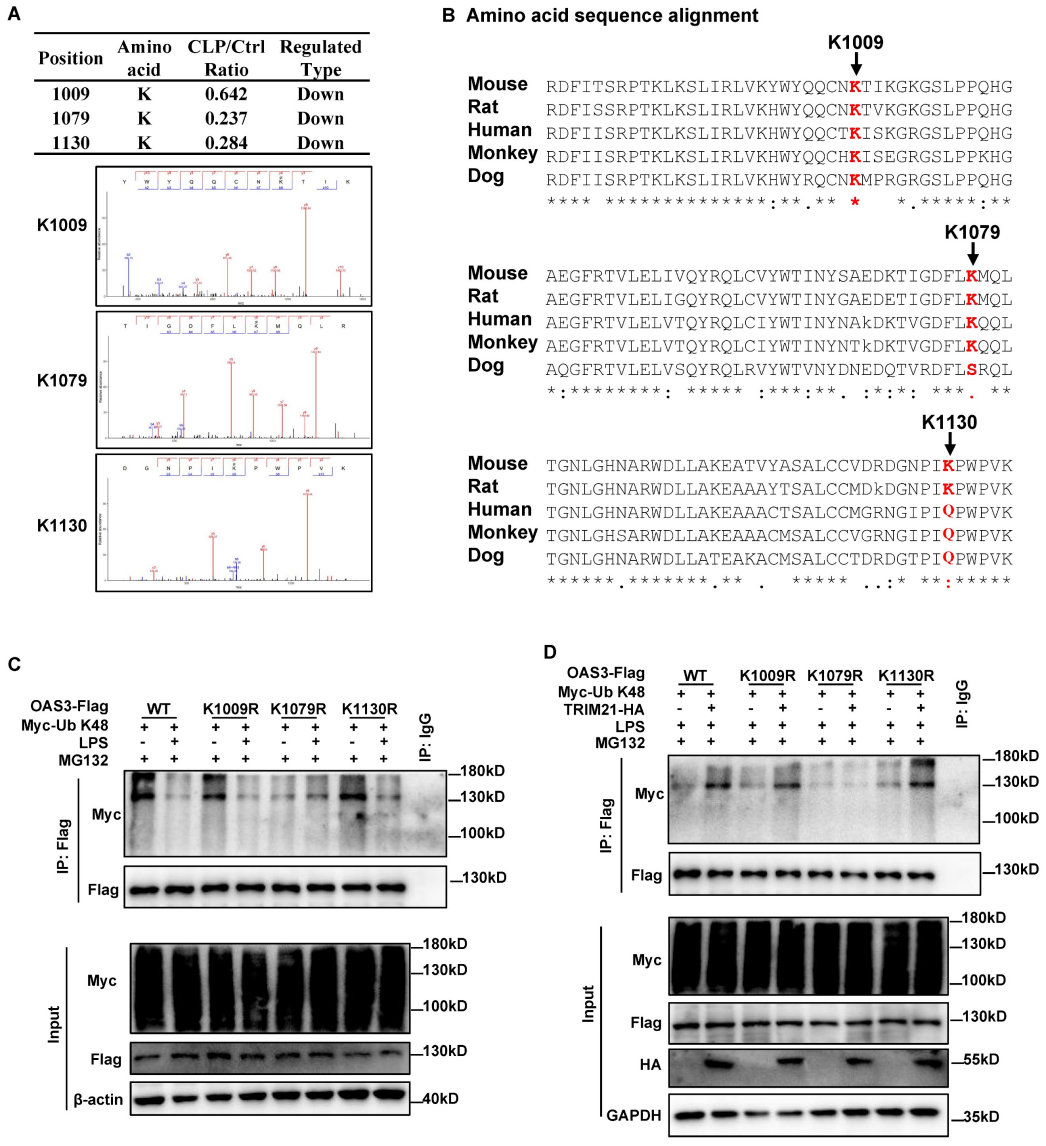
** TRIM21 mediates OAS3 ubiquitination at K1079 under LPS treatment. (A)** Mass spectrometry (MS) analysis of the three ubiquitinated OAS3 sites (K1009, K1079 and K1130).** (B)** Amino acid sequence alignment of the OAS3 K1009, K1079 and K1130 sites among OAS3 homologs from different species.** (C)** Ubiquitination level of OAS3 in MLE12 cells transfected with the indicated OAS3 site mutation overexpression plasmid followed by with or without LPS treatment plus MG132.** (D)** Ubiquitination level of OAS3 in MLE12 cells co-transfected with the indicated OAS3 site mutation and TRIM21 overexpression plasmid, followed by LPS plus MG132 treatment.

We subsequently sought to identify the site(s) of TRIM21-mediated K48-linked ubiquitination within OAS3. According to the ubiquitinome results, there were three ubiquitin-modified sites within OAS3 containing quantitative information, all of which were downregulated in the CLP group compared with the Sham group (Fig. 6A). Interestingly, all three Ub-modified sites of OAS3 detected by MS are relatively conserved among different species (Fig. 6B). We next individually mutated these three sites from lysine (K) residues to arginine (R), which cannot be ubiquitinated, and transferred the indicated site-mutated plasmids into the MLE12 cells. The OAS3 ubiquitination level did not decrease after LPS treatment in MLE12 cells overexpressed with the OAS3 K1079R plasmid, while such an effect did not appear in MLE12 cells overexpressed with the OAS3 K1009R or K1130R plasmid (Fig. 6C), indicating that LPS weakens OAS3 K48-linked polyubiquitination at K1079 sites. Meanwhile, overexpression of TRIM21 failed to increase OAS3 polyubiquitination only in MLE12 cells overexpressed with the OAS3 K1079R plasmid (Fig. 6D). Taken together, these results suggested that TRIM21 mediates OAS3 K1079 site ubiquitination under LPS treatment.

### OAS3 promotes cell apoptosis through RNase L in sepsis

**Figure 7:**
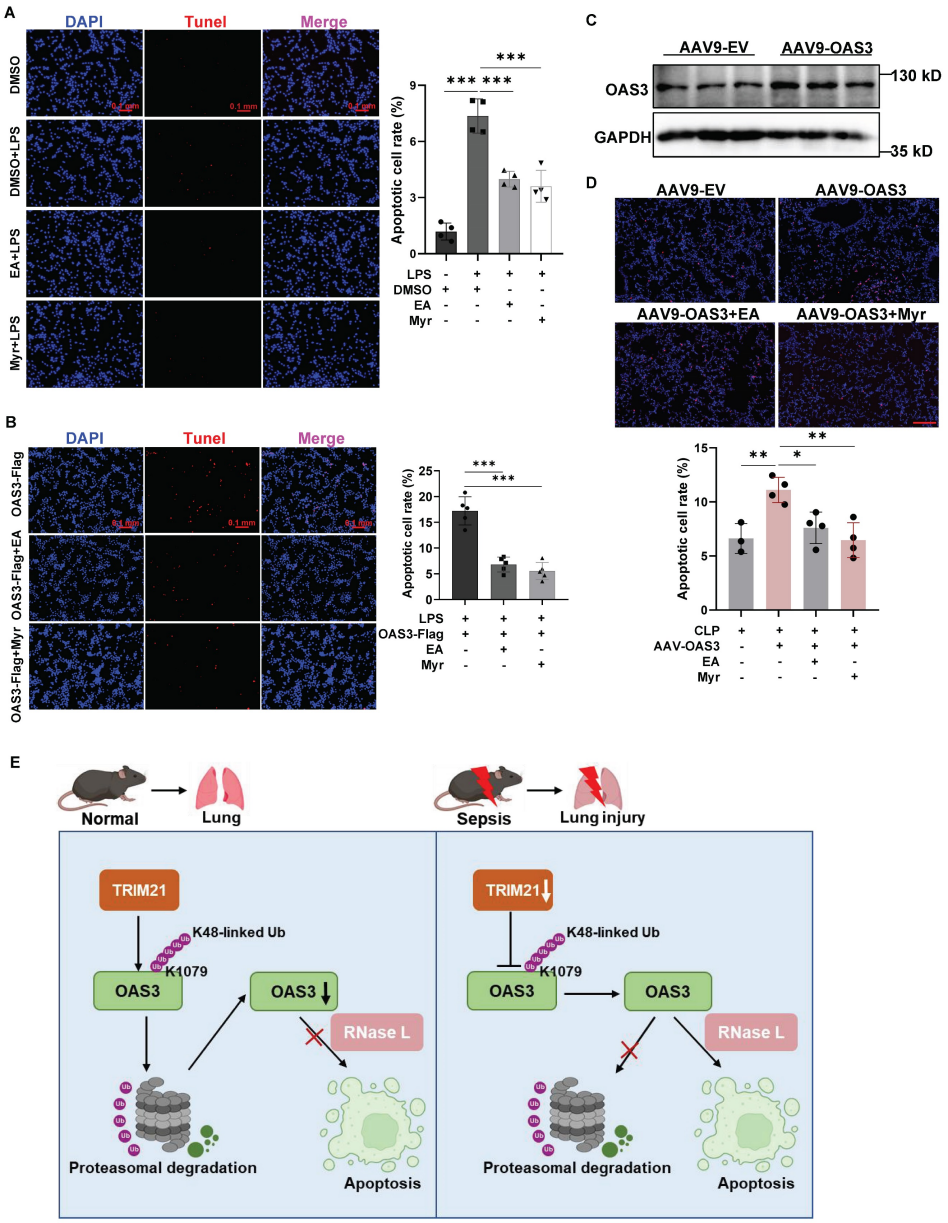
** OAS3 promotes cell apoptosis through RNase L in sepsis. (A)** TUNEL-positive cells (stained with Cy3, red) and nuclei (stained with DAPI, blue) in MLE12 cells treated with RNase L inhibitor Ellagic acid (EA) or Myricetin (Myr) plus LPS were observed and analyzed using a fluorescence microscope (Zeiss) (*n=*4, scale bar: 100 μm). **(B)** TUNEL-positive cells in MLE12 cells transfected with the OAS3 overexpression plasmid followed by RNase L inhibitor plus LPS treatment (*n=*5, scale bar: 100 μm). **(C)** WB analysis of the OAS3 protein level in lung tissue of mice intratracheally injected with the empty vector (EV) or OAS3 overexpression AAV9. **(D)** TUNEL-positive cells in lung tissue of mice intratracheally injected with the empty vector (EV) or OAS3 overexpression AAV9 followed by RNase L inhibitor plus CLP operation (*n*=3-4, scale bar: 100 μm). **(E)** Schematic diagram illustrating the function of OAS3 ubiquitination in acute lung injury due to sepsis. Data are shown as *Mean* ± *SD*. ***P<0.001.

Lastly, we focused on the potential mechanism by which OAS3 promotes lung epithelial cell apoptosis. RNase L is a known downstream molecule activated by OAS3 and participates in cell apoptosis. Ellagic acid (EA) and myricetin (Myr) are reported as inhibitors of RNase L^9^,^22^. Through the TUNEL staining assay, we observed that EA and Myr significantly reduced the LPS-induced increased apoptotic MLE12 cell number (Fig. 7A), indicating that RNase L is responsible for the LPS-induced apoptosis of lung epithelial cells. Furthermore, OAS3 overexpression-induced apoptotic cell number significantly decreased after inhibition of RNase L with EA and Myr in MLE12 cells under LPS stimulation *in vitro* (Fig. 7B) or in mice under CLP operation *in vivo* (Fig. 7C-D), suggesting that OAS3 aggravates cell apoptosis via RNase L in sepsis.

## Discussion

In this study, we identified OAS3 as a possible candidate resulting in sepsis-induced acute lung injury (SALI) through multi-omics analysis and confirmed its role as a promoter for SALI and epithelial cell apoptosis in the sepsis model. In the pathophysiological process, sepsis triggers the inflammatory cascade, inducing the apoptosis of lung epithelial cells^23^. Researchers suggest that disruption of the epithelial barrier caused by epithelial cell apoptosis may be one of the core adverse consequence of SALI^24^,^25^. Damage to epithelial-barrier integrity facilitates the accumulation of inflammatory cells and the leakage of plasma proteins into alveolar space, hence obstructing normal gas exchange^26^. Small molecules capable of selective inhibition of OAS3 may therefore have therapeutic value in inhibiting lung epithelial cell apoptosis as anti-SALI drugs.

Ubiquitination is often associated with the K48-linked chain, which directs the substrate toward proteasomal degradation^27^,^28^. In this study, we indicated that OAS3 protein increases in a proteasomal-dependent way and OAS3 K48-linked polyubiquitination decreases due to the E3 ligase TRIM21 downregulation in the sepsis model, which is consistent with the traditional degradative function of K48-linked chains. Besides the degradative function, ubiquitination also participates in non-degradative process: monoubiquitination, multimonoubiquitination and non-K48 polyubiquitination chains can drive non-degradative outcomes^5^,^27^,^29^,^30^. For example, TRIM21 ubiquitinated p65 via K63 linkage and then enhanced the interaction between p65 and IκB kinase to promote keratinocyte inflammation in psoriasis^31^. In terms of ubiquitination sites within OAS3, there are three ubiquitin-modified sites identified by mass spectrum (MS). Further validation found that LPS decreased OAS3 K48-linked polyubiquitination only at K1079 but not at the other two sites, K1009 and K1130, indicating that other types of polyubiquitination may occur at these two sites and need to be further explored.

Ubiquitination is regulated by ubiquitin E3 ligases and deubiquitinating enzymes (DUBs)^7^. For the first time, we identified TRIM21 as the E3 ligase accounting for OAS3 K48-linked polyubiquitination at the K1079 site and its dowregulation in the sepsis model led to a decrease in the OAS3 ubiquitination level and an increase in the OAS3 protein level in lung epithelial cells. Morever, it has been revealed that E3 ligase TRIM21 in lung endothelial cells exhibits a modulatory function during the inflammatory response to LPS^18^,^32^, indicating that TRIM21 may serve as potential therapeutic target for lung injury during sepsis.

OAS3 acts as a pattern-recognition receptor for dsRNA derived from cellular origin like mitochondria and exogenesis like RNA viruses^33^. In humans, there are four OAS genes, named OAS1, OAS2, OAS3 and OASL. OAS1, OAS2, and OAS3 contain one, two, and three core OAS units, respectively, which encode catalytically active proteins to synthesize 2-5A and activate RNase L^34^. OASL, containing one basic unit and two ubiquitin-like domains, does not produce 2-5A but rather activates RIG-I signaling^35^. Among the catalytically active forms of OAS proteins, OAS3 exhibited a higher affinity for dsRNA compared to OAS1 or OAS2, aligning with its dominant role in activating RNase L, indicating that OAS3 is mainly accountable for synthesizing 2-5A activators of RNase L^36^. However, this study presently only focused on the protein level of OAS3 and did not investigate its activity in depth. Considering that its activity is mediated by dsRNA, which originates from endogenous and exogenous sources, and that exogenous dsRNA was not involved in the LPS-treated cellular sepsis model used in this study, it is suggested that LPS may activate OAS3 activity by stimulating the production of endogenous dsRNA. It has been reported that LPS could produce dsRNA in mammalian macrophages and monocytes^37^, and this research has also preliminarily found the aggregation of dsRNA in lung epithelial cells with LPS treatment (data not shown), which is included in our upcoming research plans.

In pathophysiological mechanism, we confirmed that OAS3 promotes epithelial cell apoptosis through RNase L in the spetic model by using RNase L inhibitors ellagic acid and myricetin. RNase L is a metal ion-independent endoribonuclease activated by binding to 2-5A synthesized by OASs^36^, which has been widely recognized to be involved in antiviral and antitumor defenses^38^-^40^. The activated RNase L cleaves ssRNA and initiates apoptosis through JNK^41^,^42^. In this process, mitochondria undergo morphological and physiological changes, including reactive oxygen species production, cytochrome C release, and a loss of mitochondrial membrane potential^11^. These mechanisms underlying apoptosis reasonably explain the role of OAS3/RNase L in inducing lung epithelial cell death in the sepsis environment.

In conclusion, our data shed light on the relationship between OAS3 ubiquitination and SALI. Furthermore, our findings reveal a new mechanism linking OAS3 ubiquitination and lung epithelial cell apoptosis in SALI, in which downregulation of TRIM21 mediates the decrease of OAS3 K48-linked polyubiquitination at the K1079 site and the increase of OAS3 promotes cell apoptosis through RNase L (Fig. 7E). These findings provide rationale for developing drugs based on these targets and offer a new therapeutic strategy for sepsis-associated lung injury.

## Supplementary Material

Supplementary methods and figures.

Supplementary tables.
