# Supplementary material for: OAS3 Deubiquitination Due to E3 Ligase TRIM21 Downregulation Promotes Epithelial Cell Apoptosis and Drives Sepsis-induced Acute Lung Injury
Source: Int J Biol Sci. 2024 Oct 14;20(14):5594–607. doi: 10.7150/ijbs.96089 (PMC11528449; doi:10.7150/ijbs.96089)
Supplement: Supplementary file 1 — Supplementary methods and figures. [file ijbsv20p5594s1.pdf]

Supplementary Information for

OAS3 Deubiquitination Due to E3 Ligase TRIM21 Downregulation Promotes Epithelial Cell Apoptosis and Drives Sepsis-induced Acute Lung Injury

Zhenfeng Chen, Bingqi Lin, Xiaodan Yao, Yihang Fang, Jinlian Liu, Ke Song, Lina Tuolihong, Zirui Zuo, Qi He, Xiaoxia Huang, Zhuanhua Liu, Qiaobing Huang, Qiulin Xu, Zhifeng Liu, Xiaohua Guo

Supplementary Figures 1-4

Supplementary Methods

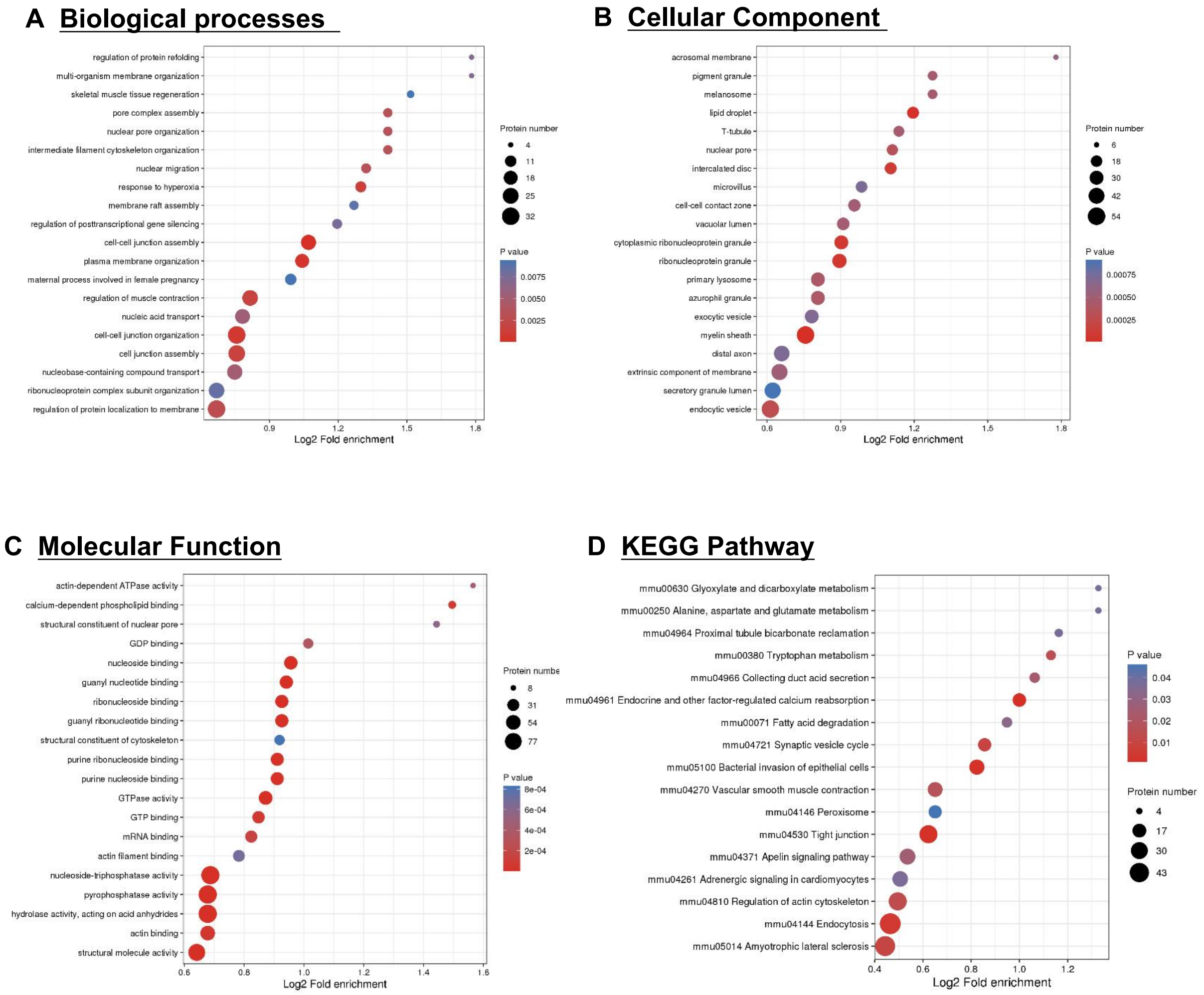

Fig. S1 Functional enrichment analysis of differentially modified proteins. (A) Biological processes of the differentially modified proteins. **(B)** Cellular component of the differentially modified proteins. **(C)** Molecular function of the differentially modified proteins. **(D)** KEGG pathway of the differentially modified proteins**.**

A

Method 1	
Ubiquitinome	Down
Proteome	Up
Transcriptome	Unchanged / Down

B

Method 2	
Ubiquitinome	Up
Proteome	Down
Transcriptome	Unchanged / Up

C

Omics	CLP vs Sham	Threshold
Ubiquitinome	Up<br>Down	FC＞1.5<br>FC＜1/1.5
Proteome	Up<br>Down<br>Unchanged	FC＞1.5<br>FC＜1/1.5<br>1.2≥FC ≥ 1/1.2
Transcriptome	Up<br>Down<br>Unchanged	FC≥2, Padj≤0.05<br>FC≤1/2, Padj≤0.05<br>-2<FC<2, Padj ≥ 0.1

D

Protein accession	Gene name	Protein description	Prontein Change_CLP/Ctrl Ratio	Position	Amino acid	Ub modification Change_CLP/Ctrl Ratio	Ub Regulated Type
Q8VI93	Oas3	2'-5'-oligoadenylate sy	6.061	1130	K	0.284	Down
Q8VI93	Oas3	2'-5'-oligoadenylate sy	6.061	1079	K	0.237	Down
Q8VI93	Oas3	2'-5'-oligoadenylate sy	6.061	1009	K	0.642	Down
Q9CQP2	Trappc2	Trafficking protein part	3.446	135	K	0.12	Down
Q9ET01	Pygl	Glycogen phosphoryla	3.229	465	K	2.185	Up
Q9ET01	Pygl	Glycogen phosphoryla	3.229	545	K	1.597	Up
Q9ET01	Pygl	Glycogen phosphoryla	3.229	170	K	1.548	Up
Q9ET01	Pygl	Glycogen phosphoryla	3.229	290	K	0.544	Down
Q61093	Cybb	Cytochrome b-245 hea	2.289	508	K	2.574	Up
Q61093	Cybb	Cytochrome b-245 hea	2.289	506	K	2.176	Up
Q61093	Cybb	Cytochrome b-245 hea	2.289	437	K	0.525	Down
Q99J72	Apobec3	DNA dC->dU-editing	1.999	295	K	0.526	Down
Q9JL16	Isg20	Interferon-stimulated g	1.848	82	K	0.467	Down
O09005	Degs1	Sphingolipid delta(4)-c	1.803	278	K	0.261	Down
Q3TBD2	Arhgap45	Rho GTPase-activating	1.78	734	K	0.321	Down
Q3TBD2	Arhgap45	Rho GTPase-activating	1.78	735	K	0.399	Down
Q3TBD2	Arhgap45	Rho GTPase-activating	1.78	131	K	0.31	Down
Q3TBD2	Arhgap45	Rho GTPase-activating	1.78	133	K	0.565	Down
Q3TBD2	Arhgap45	Rho GTPase-activating	1.78	36	K	0.355	Down
Q62415	Ppp1r13b	Apoptosis-stimulating	1.739	293	K	0.369	Down
Q9R049	Amfr	E3 ubiquitin-protein lig	1.71	353	K	0.537	Down
Q91W36	Usp3	Ubiquitin carboxyl-terr	1.705	423	K	0.563	Down
Q9JK23	Psmg1	Proteasome assembly	1.668	11	K	0.234	Down
P30681	Hmgb2	High mobility group pr	1.632	114	K	0.541	Down
Q64337	Sqstm1	Sequestosome-1 OS=l	1.551	437	K	0.629	Down
Q9ES52	Inpp5d	Phosphatidylinositol 3,4	1.549	424	K	0.655	Down
Q3U9G9	Lbr	Delta(14)-sterol reduct	1.531	188	K	0.494	Down
Q3U9G9	Lbr	Delta(14)-sterol reduct	2.531	200	K	0.377	Down
Q3U9G9	Lbr	Delta(14)-sterol reduct	3.531	50	K	0.543	Down
Q3U9G9	Lbr	Delta(14)-sterol reduct	4.531	5	K	0.623	Down
Q3U9G9	Lbr	Delta(14)-sterol reduct	5.531	612	K	0.543	Down
Q3U9G9	Lbr	Delta(14)-sterol reduct	6.531	42	K	0.61	Down
Q8VCW4	Unc93b1	Protein unc-93 homolc	1.502	582	K	0.495	Down
Q8VCW4	Unc93b1	Protein unc-93 homolc	1.502	197	K	0.599	Down

Fig. S2 (A) Method 1 parameters of multi-omics screening to identify candidate proteins. **(B)** Method 2 parameters of multi-omics screening to find candidate proteins. **(C)** Threshold for multi-omics screening methods. **(C)** Screening result of multi-omics intersection genes (Partial display).

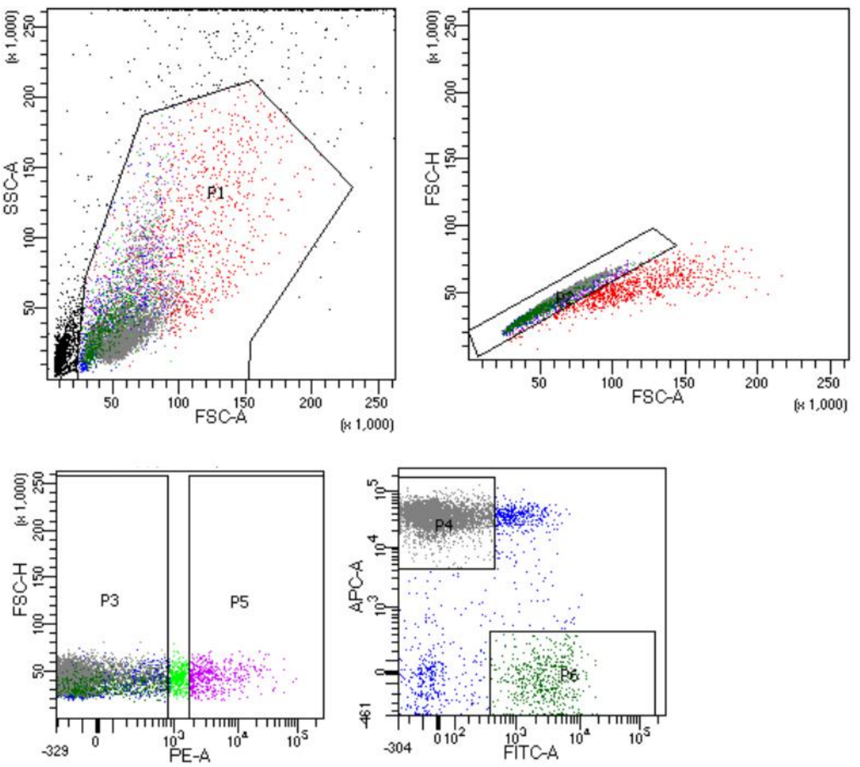

Fig. S3. Flow sorting of mouse lung epithelial cells, endothelial cells and leukocytes. P4 gate: APC-CD45 positive, indicates leukocytes. P5: PE-CD326 positive, indicates epithelial cells. P6: FITC-CD31 positive, indicates endothelial cells.

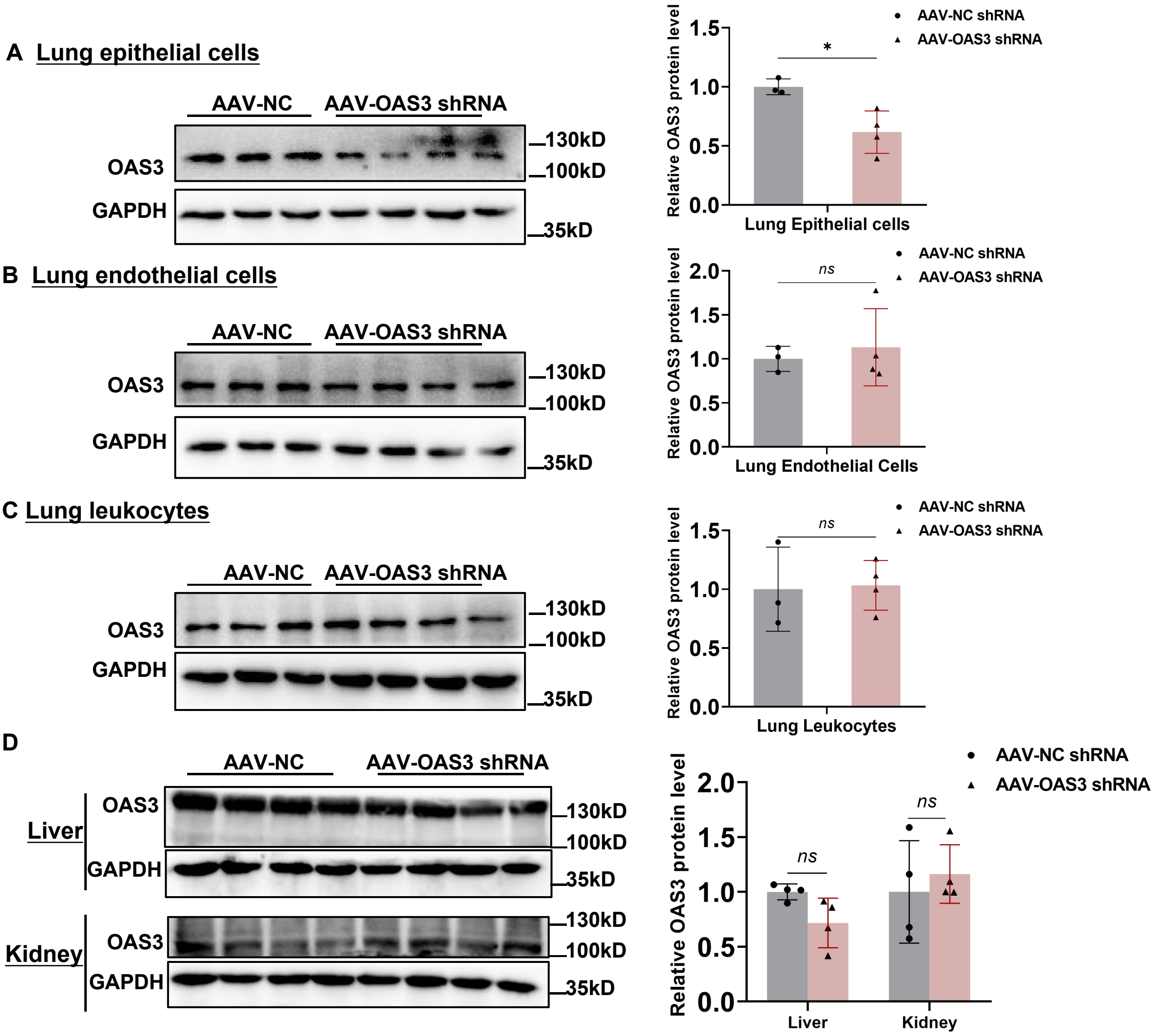

Fig. S4 Knock-down efficiency validation in mice intratracheally injected with lung epithelial cells specifically expressed AAV9-OAS3 shRNA (inserted with SFTPC promoter element). (A) OAS3 protein level in lung epithelial cells; **(B)** OAS3 protein level in lung endothelial cells. **(C)** OAS3 protein level in lung leukocytes. **(D)** OAS3 protein level in liver and kidney tissue of mice. *n*=3-4. Data are shown as *Mean* ± *SD*. **P*<0.05, ***P*<0.01, ****P*<0.001.

Supplementary Methods

Transduction of adeno-associated virus serotype 9 (AAV9) in mice

AAV9-shOAS3 and AAV9-shNC (negative control) with the SFTPC promoter (lung epithelial cells-specifically expressed) were constructed by Genechem (Shanghai, China). Mice were administrated with AAV9 viral genome particles (1.0×10^{11} viral genomes in 80 μL 0.9% saline) via intratracheal injections. Briefly, mice were anesthetized with 2% sodium pentobarbital using intraperitoneal injection, and then the neck incision was implemented. The AAV9 was injected into trachea from the tracheal cricoid cartilage of the mouse and then the incision was sutured. Subsequent experiments were performed at least three weeks after the administration.

Transfection of plasmids or siRNA

The eukaryotic expression plasmids were obtained from GENEYUAN or MiaoLingBio Corporation (Table S1). In brief, flag-tagged OAS3 site-directed lysine ubiquitination-deficient mutants (Lysine → Arginine, K → R) were constructed using the Mut Express®II Fast Mutagenesis Kit (Vazyme, Cat# C214). The ubiquitin mutant (K6, K11, K27, K29, K33, K48, or K63) contains only one indicated lysine and the remaining lysine residues were all mutated to arginine; hence, only one indicated lysine for poly-ubiquitination linkage can be formed. All constructed plasmids were sequenced, and the correct clones were selected for the further experiments. The following transfection of plasmids was performed using Lipofectamine® 2000 Reagent (Invitrogen) in accordance with the manufacturer's instruction. Briefly, MLE12 cells were cultured to 60%-70% confluence and then added with plasmid DNA-lipid complex in Opti-MEM for 48 hours, then subjected to the subsequent treatments and detections.

Transfection of siRNA was conducted following the instruction provided by GenePharma (Shanghai, China). In brief, MLE12 cells were seeded in 6-well plates with 30%–50% confluence, and transfected with specific siRNA or negative control (NC) siRNA (25 nmol/L) in serum-free, antibiotic-free endothelial cell medium containing 5 uL siRNA-Mate (GenePharma, China, Cat# G04002). The siRNA-targeted sequences are as follows: NC siRNA (sense 5'-UUCUCCGAACGUGUCACGUTT-3' and antisense 5'-ACGUGACACGUU

CGGAGAATT-3'), OAS3 siRNA (sense 5'-GCUGUUCAGUCAAAGGAAATT-3' and antisense 5'- UUUCCUUUGACUGAACAGCTT-3')

Apoptotic cell detection assay

A TUNEL assay kit (C1089, Beyotime) was used to evaluate apoptosis based on the manufacturer's instructions. In brief, an appropriate amount of TUNEL test solution (every 50μl solution contains 5μl of TdT enzyme and 45μl of Fluorescent Detection Solution) was prepared and mixed thoroughly. 50μl TUNEL solution was added to the sample and incubated at 37 ℃ for 60 min away from light. TUNEL-positive cells (stained with Cy3, red) and nuclei (stained with DAPI, blue) were photographed in five random fields (100x) under a fluorescence microscope (Zeiss) and counted with ImageJ software.

Histopathology Analysis

The lungs were flushed with 0.9% saline and excised. The left upper lobe of each mouse was removed and immersed in 4% formaldehyde solution for 24 h, then embedded with paraffin and sliced into 4 μm-thick sections. The sections were subjected to Hematoxylin-Eosin staining by standard techniques. The lung injury score was assessed based on the previous article[1]: no injury=0; injury to 25% of the field=1; injury to 50% of the field=2; injury to 75% of the field=3 and diffuse injury=4. Five visual fields were taken from each slice for lung injury scoring.

Bronchoalveolar lavage fluid (BALF) analysis

Lungs were intratracheally lavaged with 1mL pre-cold 0.9% saline for 2 times, and then the liquid was centrifuged at 3000 rpm at 4°C for 15 min. The supernatant was collected and total protein concentration was quantified by a bicinchoninic acid (BCA) kit (GBCBIO, China). The cell pellets were re-suspended with 0.2 mL pre-cold PBS and counted with a cell hemocytometer.

Flow-cytometric cell sorting

The lungs of each mouse were flushed with 0.9% saline and excised. The right lobes of each mouse were removed to a grinding tube with two grinding beads and digested in lysing buffer

(PBS + 1 mg/mL collagenase I + 0.01 mg/mL DNaseI) at 37 °C for 50 min. Then the tissue homogenates were filtered through 70 μm cell strainers (CORNING, Cat# 352350) and centrifuged at 400g for 5 min. The precipitate was lysed with 1 mL ACK (Beyotime, #C3702-500mL) for 5 min, washed once with PBS+2% FBS and centrifuged at 400g for 5 min. The cell pellets were re-suspended in 0.2 mL PBS+2% FBS and incubated with several antibodies (APC-CD45, PE-CD326 and FITC-CD31) at 4 °C for 30 min in the dark. Lastly, the cells were washed once, re-suspended in 0.5mL PBS+2% FBS and sorted immediately by flow cytometer (BD Biosciences).

Protein preparation and western blot analysis

Total proteins were extracted by using lysis buffer supplemented with phosphatase and protease inhibitor cocktail. The protein concentration was detected by a BCA kit (GBCBIO, China). Protein extracts were subjected to SDS-PAGE and transferred to polyvinylidene difluoride (PVDF, Merck Millipore, USA) membranes, which were then blocked with 5% skimmed milk for 1 h at room temperature and incubated with specific primary antibodies overnight at 4 ℃, followed by being incubated with HRP-conjugated secondary antibodies for 1 h at room temperature. The band was visualized by chemiluminescence and quantified by ImageJ.

Co-Immunoprecipitation (Co-IP)

Lung tissue of mouse or MLE12 cells were collected and lysed using Cell Lysis Buffer for Immunoprecipitation (Beyotime, China, Cat# P0013). The lysate was obtained and then incubated with OAS3 antibody, Flag tag antibody or HA tag antibody with a volume ratio of 1:100 overnight at 4 °C and then added with 20 μL Protein A+G Magnetic Beads (Beyotime, China) at 4 °C for 2 h. Beads were then washed with PBS three times and resuspended in 1×SDS sample buffer, followed by western blotting with a specific antibody against the OAS3 or Flag tag or HA tag.

Quantitative real-time PCR (qPCR)

Total RNA was prepared from MLE12 cells or lung tissues with TRIzol reagent (GBCBIO, China) under the manufacturer's protocol. Complementary DNA synthesis was performed using HiScript® III RT SuperMix (Vazyme, China). Real-time PCR was conducted in a 7500 Real-Time PCR Platform (Applied Biosystems, USA) with specific primers. The primers used for qPCR are listed as followed: *Oas3* (Forward 5'-CAAAGCGTGGACTTTGACG-3' and Reverse 5'-ATGGTCTTGTTACACTGTTGGTA-3'), *Gaphd* (Forward 5'- AGGTCGGTGTG AACGGATTTG-3' and Reverse 5'- TGTAGACCATGTAGTTGAGGTCA -3'). Data were normalized to the expression of a control gene (GAPDH) and the 2−ΔΔCt method was applied to quantify mRNA expression.

Immunofluorescent staining

MLE12 cells were plated on the glass bottom dish (Cellvis, Cat# D35-10-1-N) and cultured to 70%-90% confluence and then co-transfected with the OAS3-Flag and TRIM21-HA overexpression plasmid for 48 hours. Cells were fixed and permeabilized using 4% formaldehyde and 0.5% Triton X-100, respectively, for 10 minutes, subsequently blocked with 5% BSA and incubated with primary antibodies targeting Flag tag and HA tag overnight at 4 ℃. Finally, the nucleus was stained with DAPI (1:1000) and images were captured with the laser confocal scanning microscope (Zeiss LSM780, Germany).

References:

1. Su X, Wang L, Song Y, Bai C. Inhibition of inflammatory responses by ambroxol, a mucolytic agent, in a murine model of acute lung injury induced by lipopolysaccharide. Intensive Care Med. 2004; 30: 133-40.
